# Cost-effectiveness analysis of operative versus non-operative management of colorectal cancer metastases in the Finnish RAXO Study

**DOI:** 10.2340/1651-226X.2026.45005

**Published:** 2026-02-03

**Authors:** Joel Kontiainen, Kaisa Lehtomäki, Timo Muhonen, Jarmo Hahl, Iiro Toppila, Tuija Poussa, Emerik Osterlund, Eetu Heervä, Hanna Stedt, Raija Kallio, Päivi Halonen, Arno Nordin, Aki Uutela, Tapio Salminen, Sonja Aho, Maarit Bärlund, Annika Ålgars, Raija Ristamäki, Annamarja Lamminmäki, Bengt Glimelius, Helena Isoniemi, Pia Osterlund

**Affiliations:** a Department of Oncology, TAYS Cancer Centre, Tampere University Hospital, Wellbeing Services County of Pirkanmaa, Tampere, Finland; b Faculty of Medicine and Health Technology, Tampere University, Tampere, Finland; c Department of Oncology, Helsinki University Hospital and University of Helsinki, Helsinki, Finland; d Medaffcon Ltd, Espoo, Finland; e StatConsulting, Nokia, Finland; f Department of Oncology, Turku University Hospital and University of Turku, Turku, Finland; g Department of Immunology, Genetics and Pathology, Uppsala University, Uppsala, Sweden; h Department of Oncology, Kuopio University Hospital and University of Eastern Finland, Kuopio, Finland; i Department of Oncology, Oulu University Hospital and University of Oulu, Oulu, Finland; j Transplantation and Liver Surgery Unit, Abdominal Centre, Helsinki University Hospital and University of Helsinki, Helsinki, Finland; k Department of Oncology and Pathology, Karolinska Institutet and Karolinska University Hospital, Stockholm, Sweden

**Keywords:** Cost-effectiveness analysis, colorectal neoplasms, metastasectomy, quality-adjusted life years, health care costs

## Abstract

**Background and purpose:**

Cancer therapies place an increasing financial burden on societies. In metastatic colorectal cancer (mCRC), an optimised curative-intent treatment combines metastasectomy, local ablative therapy, and perioperative systemic anti-cancer therapy (SACT) under multidisciplinary team guidance. The resource-intensive operative treatment strategy results in better survival than a non-operative approach with SACT only. The cost-effectiveness of the strategy including operative treatment has not been investigated in the era of modern treatment options.

**Patient/material and methods:**

A Markov model was developed to estimate lifetime healthcare costs and quality-adjusted life-years (QALYs). Patients receiving operative treatment, including metastasectomy along with SACT, and those receiving non-operative treatment with SACT only, were identified from the prospective Finnish RAXO study that recruited 1,086 patients between 2012 and 2018. Cost-effectiveness analyses and sensitivity analyses were conducted from the healthcare payer’s perspective using 2023 cost levels.

**Results:**

The mean lifetime costs (158,309€) for patients with an operative treatment produced 6.57 life years and 5.91 QALYs according to the Markov model. The non-operative treatment group had costs of 77,182€, producing 1.99 life years and 1.74 QALYs. The incremental cost-effectiveness ratio (ICER) was 19,455€/QALY, with the caveat that more favourable characteristics were present in the operative group. In probabilistic sensitivity analyses with a willingness-to-pay threshold of 30,000€/QALY, the operative treatment group had an 81% probability of being cost-effective. The results were robust in adjusted sensitivity analyses, including propensity score matched subgroups.

**Interpretation:**

An operative treatment strategy is cost-effective at a commonly referenced acceptability threshold.

## Introduction

Colorectal cancer (CRC) is the third most common cancer and the second leading cause of cancer mortality with 0.9 million annual deaths globally [1]. By 2040, the incidence is expected to grow from the current 1.9 million to 3.1 million new cases per year [2]. At diagnosis, 20%–25% of patients have synchronous metastases, and 15%–20% will develop metastatic CRC (mCRC) later [3, 4].

An operative treatment strategy, combining metastasectomy, local ablative therapy (LAT), and/or perioperative systemic anti-cancer therapy (SACT), has demonstrated 5-year overall survival (OS) rates of 44%–66% in patients with liver, lung, and other metastatic sites [3, 5–7]. Repeated resectability assessments generate high metastasectomy rates [6, 8–10]. When a curative-intent operative treatment is not possible, non-operative treatment with palliative SACT aiming for life-prolongation and health-related quality-of-life (HRQoL) improvements is used. The emergence of new treatments has led to improved survival in both operative and non-operative management [11–14].

Cancer therapies place an increasing financial burden on societies, making it essential to justify resource use based on treatment outcomes [15, 16]. Treatments requiring extensive surgical resources should be critically evaluated, as the operating room is typically the most expensive hospital facility [17]. Therefore, demonstrating the cost-effectiveness of therapies for mCRC that involve major surgeries is essential. To date, a few studies have reported that resection of liver metastases is cost-effective compared with non-operative management with an incremental cost-effectiveness ratio (ICER) < 35,000€ per quality-adjusted life-year (QALY) [18–20]. ICER < 35,000€/QALY is considered cost-effective according to National Institute for Health and Care Excellence (NICE) guidelines [21]. However, previous analyses have focused on the cost-effectiveness of optimised cohorts with upfront resectable single-site metastases without integrating the effect of modern conversion and perioperative SACT, or the role of several resections for multisite metastases. The more aggressive operative approach leads to more relapses and inferior survival but still the only possibility for cure. Therefore, the cost-effectiveness of up-to-date treatment strategies in mCRC that also include conversion therapy and repeated interventions remains unknown.

In this study, lifetime healthcare costs and QALYs were used as the primary endpoints to assess the cost-effectiveness of operative management in mCRC patients. The model inputs (e.g. survival, HRQoL, and costs) were derived from the prospective nationwide Finnish RAXO study. Sensitivity analyses including propensity score matching were used to balance for inequities between the two groups, as no randomised data are available.

## Material and methods

### Patient cohorts

The RAXO study aimed at maximised resectability through repeated assessment of technical resectability by an experienced multidisciplinary team. The study included adult consenting mCRC patients with metastases at any site who were deemed fit for anticancer treatment. In this study, 1,086 mCRC patients from the RAXO study were included at all 21 hospitals treating cancer in Finland. The setting and details for inclusion, resectability, and decision-making at the local hospital have been published in detail [6], and are summarised in Supplementary Figure 1.

Patients were allocated to the operative group if a curative-intent metastasectomy and/or LAT was performed during the disease trajectory. All other patients were allocated to the non-operative group, all of whom were fit for SACT but clinical condition deteriorated in 23 (2%) patients and they received no active tumour controlling therapy.

### Model structure

A Markov model was created to estimate lifetime healthcare costs and QALYs for patients in the two treatment groups (Supplementary Figure 2). A lifetime time-horizon was used, that is, all patients end up in the state of death. In the model, all patients began at mCRC diagnosis and, at the end of each model cycle of 30 days, stayed in the current state or moved to another health state according to the state-dependent transition probabilities. During a model cycle, patients spent healthcare resources and gained QALYs according to the properties of the current health state. The model was developed following the Professional Society for Health Economics and Outcomes Research and Society for Medical Decision Making guidelines [22]. No formal economic analysis plan was developed for this study.

Six mutually exclusive health states for RAXO data were defined and presented in detail in Kontiainen et al. [23]: diagnostic, curative treatment of metastases (denoted curative), remission, palliative SACT without metastasectomies/LAT (designated palliative), treatment break, and end-of-life (Supplementary Figure 2) [23]. Patients enter via the diagnostic health state, that comprises the time from mCRC diagnosis to curative, palliative SACT, or end-of-life. The curative state is the time during metastasectomy/LAT and/or conversion/neoadjuvant/adjuvant SACT, and the postoperative 6-month period, and thus also includes complications, deaths, and their effect on HRQoL, costs etc. The remission state constitutes the disease-free time. The palliative SACT state constitutes the time in SACT not associated with a metastasectomy/LAT. A treatment break refers to a period of over 2 months without palliative SACT. The end-of-life state is the last, up to 3 months before death, with no active cancer therapies.

### Model input parameters

To estimate transition probabilities, healthcare costs, and HRQoL for the health states, each day in the trajectory of individual patients in the RAXO study was categorised into one of the six health states. Individual input parameter data were then attached to the health states according to the categorisation of the index date.

Transition probabilities between health states are presented in Supplementary Table 1. Follow-up started at the date of mCRC diagnosis and ended at death or the cut-off date, August 18, 2023, based on updated RAXO survival data [6]. Survival beyond the observed follow-up was extrapolated using a parametric exponential model. The selection of the extrapolation model is described in Supplementary Figure 3.

A transition probability from state A to state B during a model cycle was calculated by dividing the sum of all state A to state B transitions by the sum of all state-to-state transitions from state A, including transitions back to state A. Transition probabilities were calculated from all transitions occurring during follow-up period. If a patient entered a given health state multiple times, all occurrences of that state were included in the analysis.

Cross-sectional HRQoL was collected for 93% of eligible patients during 2017–2023, in total 444 (41% of 1,086) RAXO patients. Data are presented in Supplementary Table 2. The methods for data collection and main results have been reported in detail in Lehtomäki et al [24]. We used the EuroQol five-dimension three-level questionnaire (EQ-5D-3L) health state index, which ranges from 0.00 (dead) to 1.00 (perfect health). HRQoL data were re-analysed for the present study according to the designated health states*.* As a Finnish EQ-5D-3L value set based on time trade-off methodology was not available for the estimation of utility values, the corresponding Swedish methodology was applied [25]. No adjustment for baseline HRQoL was applied in the estimation of utility values. Missing values were excluded without imputation.

In Finland, cancer care is provided within a tax-financed public healthcare system. Costs from a specialist healthcare provider perspective were collected for 941 (88% of 1,086) patients from the six largest hospitals. The costs for the health states used in this study have been described, and the generalisability of the costs has been discussed in detail in Kontiainen et al. [23]. These medical expenses include all specialist healthcare costs, whether directly associated with mCRC or not. The non-hospital pharmacy costs for oral SACT drugs, such as capecitabine, were estimated based on the actual utilisation reported in the study above [23]. The cost data did not include primary care costs, which were added based on estimations from a study investigating costs, HRQoL, and resource usage for Finnish CRC patients in 2009–2011 [26]. Out-of-pocket payments, around 3% of total costs in Finnish patients with metastatic cancer [27], were not available for the analysis. Finally, all costs were adjusted to 2023 price levels [28]. A breakdown of the monthly costs for each health state is presented in Supplementary Table 3.

### Model assumptions

Transition probabilities were estimated separately for the operative and non-operative groups throughout the treatment trajectory, but costs and HRQoL were assumed to be the same for all patients in a health state regardless of treatment group (i.e. the cost for one cycle of palliative SACT state was the same whether the patient previously underwent a metastasectomy or not). A beta distribution was assumed for transition probabilities in states with two transition options, and a multivariate beta distribution (Dirichlet) for states with more than two transition options. A gamma distribution was assumed for HRQoL distributions and healthcare costs [29]. Future costs, QALYs, and life-years were discounted at a 3% annual rate, as recommended [30]. A half-cycle correction was used for both costs and utilities.

### Analysis of data

Five analyses were conducted to calculate cost-effectiveness and assess the uncertainty. First, lifetime costs, QALYs, and ICER were estimated using a base-case analysis, which presents the most likely outcome of the intervention by using the most plausible set of input values. Second, probabilistic sensitivity analyses (PSA) using a Monte Carlo analysis with 10,000 iterations was performed to assess the effect of uncertainty caused by the distribution of input variables. Third, a willingness-to-pay acceptability curve was produced to assess cost-effectiveness across a range of possible willingness-to-pay thresholds. Fourth, a deterministic one-way sensitivity analysis was conducted to estimate the impact of different scenarios on the results. Analyses with propensity score-matched (PSM) patient subgroups were conducted as part of one-way sensitivity analyses. The PSM was used to reduce potential confounding caused by unbalanced baseline covariates between the operative group and the non-operative group. Multivariable logistic regression was used to generate propensity scores for all patients. Two PSM scenarios were developed. For scenario 1, the logistic regression model incorporated the following categorical covariates: age, Eastern Cooperative Oncology Group performance status (ECOG PS), primary tumour location, number of metastatic sites, mutational status, and baseline resectability by central assessment characterised as upfront resectable, borderline and unresectable. For scenario 2, the number of covariates was reduced to four: ECOG PS, primary location, metastatic sites, and mutational status. The categories are described in Supplementary Table 4 and boxplots of the propensity score distributions by treatment group are presented in Supplementary Figure 3 and Supplementary Figure 4. For both scenarios, the PSM was performed using a greedy algorithm with a 1:1 ratio without replacement and a caliper width of 0.2.

Model outcomes were validated by comparing the model-derived OS with observed OS in the RAXO study. The Markov model was created and analysed using Treeage Pro software (version 2024) [31].

Continuous variables are presented as median with interquartile range (Q1–Q3), or as mean and standard deviation (SD). Counts and percentages are shown for categorical variables. Follow-up and OS durations were estimated using the reverse Kaplan-Meier method and reported as median and 95% confidence interval (CI). Standardised mean differences (SMDs) were used to assess differences between operative and non-operative groups at baseline and after PSM. For binary variables, the SMD was calculated as the difference in proportions divided by the pooled variance. For multi-category variables, SMDs were calculated and reported for each category. SMDs more than 0.10 were considered meaningful. Statistical analyses were conducted in R Statistical Software (Version 4.3.1) and IBM SPSS Statistics (Version 28) [32, 33].

The manuscript was prepared according to CHEERS 2022 standards (Supplementary Table 5) [34].

## Results

### Patient cohorts and model validation

The RAXO study included 1,086 patients between 2012 and 2018. For this study, 399 (37%) patients were allocated to the operative and 687 (63%) to the non-operative treatment group (Supplementary Figure 1). An R0–1 metastasectomy ± LAT was performed in 326 patients, an A0–1 LAT was performed in 19 patients, whereas non-radical R2 surgery was performed in 54 patients. A detailed description of the operative treatments and SACT regimens is provided in the main RAXO report [6]. At data cut-off, 241 (60%) patients had died in the operative group and 662 (96%) in the non-operative group. The median follow-up was 94 months (95% CI: 89–97) in the operative group and 90 months (95% CI: 77–112) in the non-operative group. Baseline demographics are presented in Table 1. Patients in the operative group were younger, had better ECOG PS, were more likely to have left-sided primaries, and more often presented with only one metastatic site compared with those in the non-operative group.

**Table 1:** Patient characteristics in the RAXO study.

Variable		All patients				
		N = 1,086				SMD
		Operative		Non-operative		
		*n* = 399	%	*n* = 687	%	
Follow-up, months	Median (95% CI)	93.6	(89–97)	89.7	(77–112)	
Age, years	Median (range)	65.0	(25–84)	67.4	(24–90)	
Age	≤ 70	290	73	425	62	0.23
	> 70	109	27	262	38	
Sex	Male	242	61	414	60	0.01
	Female	157	39	273	40	
ECOG	PS 0	159	40	136	20	0.45
	PS 1	210	53	390	57	-0.08
	PS 2–3	30	8	161	23	-0.45
Charlson comorbidity index	No	320	80	514	75	0.13
	1 to 2	77	19	167	24	-0.12
	3 to 5	2	0.5	6	0.9	-0.05
Smoking status	Never smoker	160	57	244	50	0.14
	Ex-smoker	83	30	178	36	-0.15
	Smoker	38	14	68	14	-0.01
BMI, kg/m2	< 20	31	8	53	8	0.00
	20–30	278	70	524	76	-0.15
	≥ 30	90	23	110	16	0.17
Primary location	Right colon	86	22	224	33	-0.25
	Left colon	176	44	220	32	0.25
	Rectum	137	34	237	34	0.00
	Multiple	0	0	6	0.9	-0.13
Surgery of primary ever	Upfront	324	81	401	58	0.51
	During	69	17	38	6	0.38
	No	6	2	248	36	-0.99
Presentation of metastases	Synchronous	231	58	505	74	-0.33
	Metachronous	168	42	182	26	
Metastatic sites	1 site	309	77	277	40	0.81
	2 sites	62	16	257	37	-0.51
	3 to 6 sites	28	7	153	22	-0.44
Mutational status	RAS +/- BRAF wt	175	44	254	37	0.14
	RAS mt	200	50	343	50	0.00
	BRAF mt	16	4	78	11	-0.28
	Not tested	8	2	12	2	0.02
Mismatch repair status	Proficient (MSS)	229	57	253	37	0.42
	Deficient (MSI-H)	8	2	10	1.5	0.04
	Not tested	162	41	424	62	-0.43
Upfront resectability by central assessment	Upfront resectable/neoadjuvant	265	66	45	7	1.58
	borderline/conversion	125	31	54	8	0.62
	Non-resectable	9	2	588	86	-3.09

SMD: standardised mean difference; BMI: body mass index; ECOG: Eastern Cooperative Oncology Group; PS: performance status; 95% CI: 95% confidence interval.

**Figure 1:**
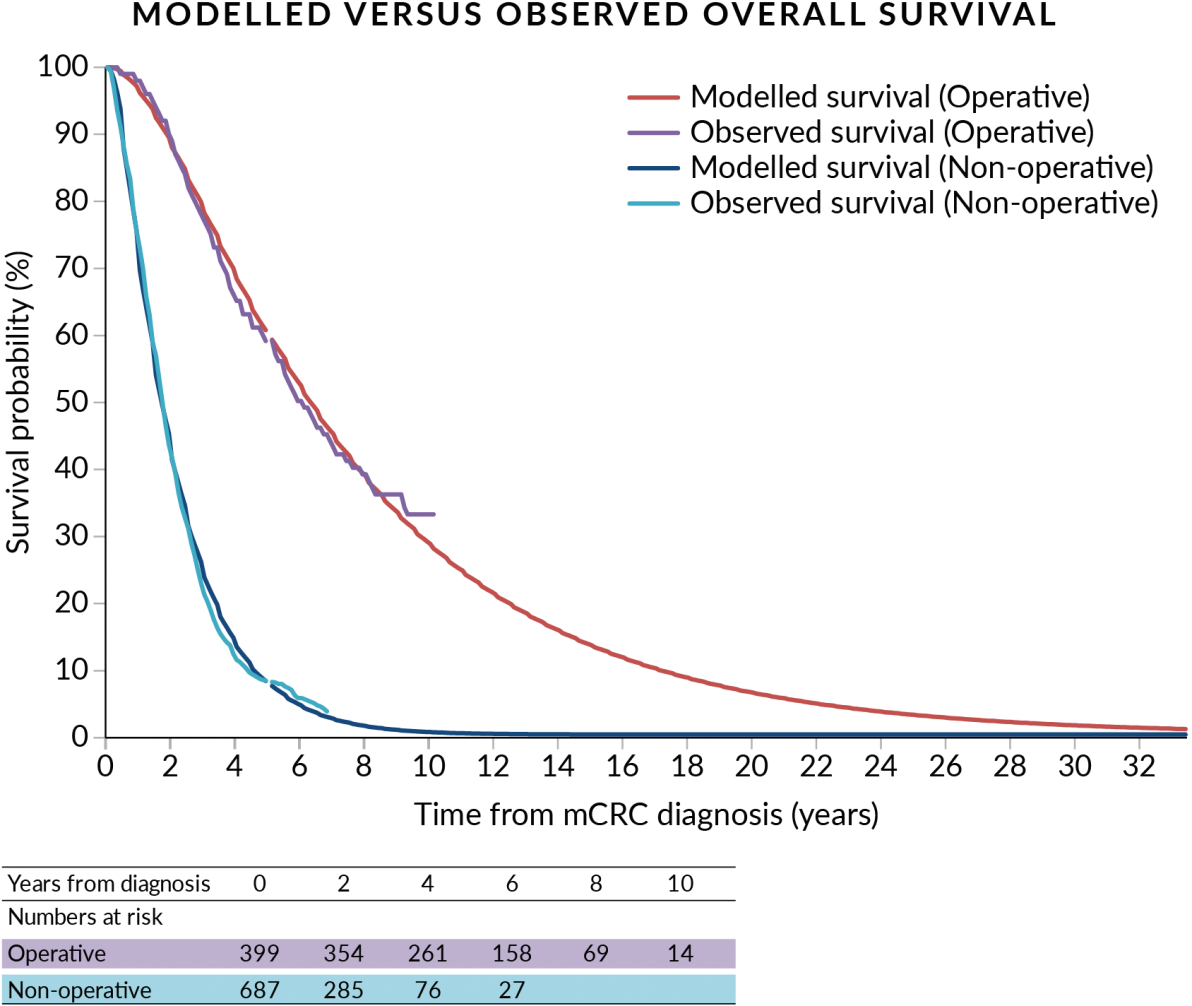
Modelled versus observed overall survival.

In the model validation analysis, the observed OS for RAXO patients was similar to the modelled OS (Figure 1)*.* For patients in the operative group, the observed median OS Kaplan-Meier estimate was 71 (95% CI: 63–79) months compared to median 73 months in the modelled base-case analysis. For patients in the non-operative group, the observed median OS was 20 (95% CI: 19–22) months compared to median 20 months in the base-case analysis.

### Costs and health utilities

The base-case analysis results are presented in Table 2. Patients with operative treatment had a mean 158,309€ lifetime healthcare cost, whereas patients with non-operative treatment had a mean cost of 77,182€ (incremental cost 81,127€). For patients in the operative treatment group, 46% of lifetime costs accumulated during the curative health state (73,094€) and 15% during the remission state (23,197€). The costs for the palliative SACT state were 49,755€ (accounting for 31%) in the operative group and 64,781€ (accounting for 84%) in the non-operative group. Estimated costs for diagnostic, treatment break, and end-of-life phases did not markedly differ between the groups, and were approximately 6,800€, 2,000€, and 3,500€, respectively. Cumulative costs and QALYs for the groups are presented in Supplementary Table 6.

**Table 2:** Base-case analysis.

Outcome	Operative		Non-operative	
	Mean	%	Mean	%
Lifetime cost, €	158,309	100	77,182	100
Diagnostic	6,814	4	6,742	9
Curative	73,094	46	-	-
Remission	23,197	15	-	-
Palliative SACT	49,755	31	64,781	84
Treatment break	2,070	1	2,047	3
End-of-life	3,380	2	3,612	5
QALY	5.91	100	1.74	100
Diagnostic	0.12	2	0.12	7
Curative	1.55	26	-	-
Remission	2.93	50	-	-
Palliative SACT	1.03	17	1.34	77
Treatment break	0.21	4	0.21	12
End-of-life	0.07	1	0.07	4
Life years	6.57		1.99	
Incremental lifetime cost, €	81,127		-	
Incremental effectiveness, QALY	4.17		-	
ICER, €/QALY	19,455		-	

QALY: quality adjusted life year; ICER: incremental cost-effectiveness ratio; SACT: systemic anti-cancer therapy.

The mean life-years reached were 6.57 years in the operative group and 1.99 years in the non-operative group. When adjusted for HRQoL, the mean QALY was 5.91 years for the operative treatment group and 1.74 years for the non-operative treatment group. Thus, the incremental QALY was 4.17 (Table 2).

The incremental lifetime costs and QALYs for patients who received operative treatment compared with non-operative treatment were 81,127€ and 4.17 QALY, respectively, resulting in an ICER of 19,455€/QALY.

### Sensitivity analyses

The PSA results are shown in Supplementary Table 7. In a Monte Carlo simulation with 10,000 iterations, the mean (SD) lifetime cost estimation for the operative treatment group was 158,964€ (67,014€), closely aligning with the 158,309€ in base-case analysis. For the non-operative group, the mean cost estimation was 77,228€ (35,736€), and 77,182€ in base-case analysis. The QALYs were 5.92 (0.42) in Monte Carlo simulation compared with 5.91 in the base-case study, and 1.74 (0.16), compared with 1.74, respectively.

**Figure 2:**
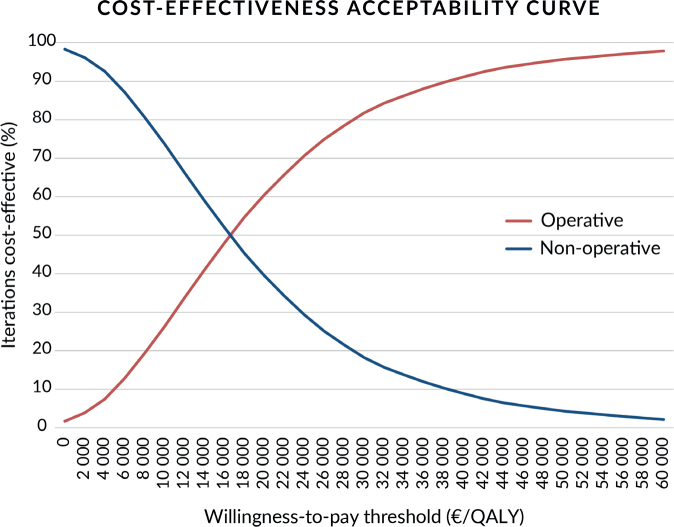
Cost-effectiveness acceptability curve.

A cost-effectiveness acceptability curve is presented in Figure 2. At a willingness-to-pay threshold of 30,000€/QALY, the operative treatment group has an 81% probability of being cost-effective and exceeds 90% at 40,000€/QALY.

In one-way sensitivity analyses, the ICER for operative versus non-operative treatment ranged between 14,330 and 37,064€/QALY (Table 3). The results were robust for changes in patient characteristics and survival. In a scenario with 50% higher mortality in operatively group in the remission health state, ICER increased to 23,400 €/QALY. In analyses with PSM cohorts, the ICER was either 18,381€/QALY (scenario 1, *n* = 175 including baseline resectability along with the five strongest prognostic factors) or 19,081€/QALY (scenario 2, *n* = 556) depending on whether resectability of metastases and age were included as matching variables or not (Table 3)*.*

**Table 3:** One-way sensitivity analyses.

Scenario type	Scenario	Group	Cost, €	QALY	ICER, €/QALY
Base-case	Base-case	Operative	158,309	5.9	19,455
		Non-operative	77,182	1.7	
Cost adjustments	Palliative SACT health state cost – 50%	Operative	133,766	5.9	21,483
		Non-operative	44,182	1.7	
	Curative health state cost +100%	Operative	231,737	5.9	37,064
		Non-operative	77,182	1.7	
	Systemic cancer drug costs – 50%	Operative	138,186	5.9	18,428
		Non-operative	61,343	1.7	
	Systemic cancer drug costs +50%	Operative	179,100	5.9	20,632
		Non-operative	93,063	1.7	
	Discount rate 5%	Operative	145,499	5.3	19,393
		Non-operative	74,907	1.7	
	Discount rate 0%	Operative	184,876	7.1	19,745
		Non-operative	81,016	1.8	
Transition probability adjustments	Morbidity (transition rate to palliative SACT, EoL and Death states) in remission health state +50%	Operative	144,797	4.6	23,396
		Non-operative	77,182	1.7	
	Mortality (transition rate to EoL and Death state) in palliative chemotherapy health state – 50%	Operative	181,876	6.4	17,422
		Non-operative	115,149	2.6	
Patient cohort adjustments	Only single-organ metastases patients	Operative	159,858	6.4	16,861
		Non-operative	84,993	2	
	Non-operative cohort including ECOG 0 and CCI 0 patients only	Operative	158,309	5.9	14,330
		Non-operative	111,020	2.6	
	Propensity score matched patient cohorts, scenario 1*	Operative	141,559	4.9	18,381
		Non-operative	91,196	2.2	
	Propensity score matched patient cohorts, scenario 2*	Operative	155,609	5.5	19,081
		Non-operative	91,688	2.1	
	Operative cohort including R0–1 operated patients only	Operative	167,829	6.7	18,239
		Non-operative	77,182	1.7	
Combination	Non-operative cohort including ECOG 0 and CCI 0	Operative	158,309	5.9	28,854
	Patients only, with Palliative SACT cost – 50%	Non-operative	63,092	2.6	

QALY: quality-adjusted life-year; ICER: incremental cost-effectiveness ratio; SACT: systemic anti-cancer therapy; EoL: End-of-life phase; ECOG: Eastern cooperative oncology group; CCI: Charlson comorbidity index.

* Propensity score matching was in scenario 1 performed based on ECOG, number of metastatic sites, mutational status, primary tumour location, resectability (upfront, borderline, or nonresectable), and age. In scenario 2, matching was done with same parameters excluding resectability and age. Details of the cohorts are provided in Supplementary Table 4 and Supplementary Figure 3.

## Discussion and conclusion

This study presents a cost-effectiveness analysis comparing operative and non-operative treatment strategies for mCRC, using data derived from a recent prospective intervention study. The results indicate that the operative treatment strategy is cost-effective, with ICER of 19,500€ per QALY. This is well below the commonly accepted cost-effectiveness thresholds of 24,000€–35,000€/QALY with NICE [21] or 38,000€/QALY as one proposed Finnish estimate [35]. The results remained robust across extensive sensitivity analyses, including propensity score matching.

Previous cost-effectiveness analyses of mCRC treatments have similarly shown that operative treatment may be cost-effective over non-operative treatment with ICERs up to 16,300€/QALY [18–20]. However, these studies did not capture the role of modern conversion/perioperative SACT, as the patient cohorts in previous studies were collected over 20 years ago. For example, in a study conducted by Roberts et al. [18] from 1992–2001, the median OS for operated patients was 41 months compared with 71 months in the current study [6]. These differences in survival emphasise the crucial role of modern active assessment of operability, high re-resection rate, and conversion/perioperative SACT given [6]. In the RAXO study, this strategy enabled surgery in 37%, and conversion in 18% of baseline borderline or unresectable tumours [6, 36]. Resection rates seem to be lower outside specialised hospitals [37]. Therefore, centralised assessment of resectability should be implemented whenever possible. The cost-effectiveness of conversion/perioperative SACT represents a direction for future research.

The strength of this study is the use of individual patient data, including clinical events, costs, HRQoL, and survival data with long follow-up from the same prospective study to calculate model input parameters. Real-life patients with multiple and multisite metastases reflecting the background population were included. It was possible to generate six health states [23], reflecting distinct health utilities in different phases of the disease trajectory, as recommended [22]. Sensitivity analyses were possible using PSM patient cohorts. Matching was based on factors known to influence resectability and OS in patients with mCRC, including age, sex, ECOG, mutational status, number of metastatic sites, and primary tumour location. Resectability of metastases was the most critical covariate, as its exclusion from the PSM analysis allowed for substantially larger group sizes but introduced significant imbalance with respect to this covariate. Age also had some impact. To assess the effect of these trade-offs, two scenarios were tested: one with and one without resectability and age. However, no meaningful differences in ICER were observed.

There are limitations to this study. First, assumptions regarding the input parameters were made. For practical reasons and model simplicity, an exponential parametric model was used for extrapolation. The resulting assumption of constant transition probabilities may introduce bias, as discussed in Supplementary Figure 3. However, given the long follow-up and the predominance of empirically observed survival data (Figure 1), the net impact of any extrapolation model is expected to be limited. Furthermore, we noted no differences in HRQoL for palliative SACT, treatment-break, and end-of-life health states or symptom scales in operative versus non-operative groups (data not shown). Therefore, they were assumed to be equal in both groups. Thus, cost inputs were assumed in line, irrespective of the treatment group, which may introduce bias. Some clinically important events, such as complications, are not modelled through distinct health states but are reflected indirectly through cost and HRQoL inputs derived from real-world data. To assess the impact of these assumptions, one-way sensitivity analyses were performed, showing that the results are robust to changes in morbidity, mortality, or costs in solitary health states. Even with a 50% increase in morbidity of patients in the remission state, the ICER does stays around 23,000 €/QALY.

A second limitation is that the cost-effectiveness of operative versus non-operative treatment groups should preferably be modelled using data from randomised studies. However, no randomised studies, apart from the Pulmonary Metastasectomy in Colorectal Cancer (PulMiCC)-study with all its limitations [38], are available, as the setting is considered unethical. Therefore, as in previous studies [18–20], this study had to rely on observational data. The two groups formed were one where surgery was performed and another where this was not possible even if the intention in many patients was ‘conversion’. In studies using non-randomised cohorts for economic modelling, propensity score matching or selecting only patients theoretically fit for the intervention in the comparison group have been applied to make the groups comparable [18, 39]. When two stringently propensity score matched, patient cohorts were used as a basis of the Markov model; the ICER remained around 19,000€/QALY. Furthermore, the ICER slightly decreased when only the fittest patients were selected for the non-operative cohort, as the costs for the palliative SACT health state increased.

Third, the study included only Finnish patients. The results may thus not be directly applicable to other countries. The treatment protocol in the RAXO study [6], which aligns with and is a basis for recent international guidelines, was actively followed in all Finnish hospitals [40–42]. The cost differences between hospitals are estimated to be low. Furthermore, the Finnish healthcare sector’s prices and public spending on healthcare are close to the Organisation for Economic Co-operation and Development (OECD) mean [43]. Therefore, the results of this study may serve as a reasonable approximation of cost-effectiveness in other developed Western countries where active metastasectomy and/or LAT are routinely used.

Fourth, cross-sectional HRQoL data collected from mid-study may induce bias. These limitations are discussed in Lehtomäki et al. [24]. In addition, utility values were not adjusted for baseline HRQoL. As is common in HRQoL studies, 16% of questionnaire data were missing, which may introduce bias if missingness is related to treatment toxicity or other HRQoL-related circumstances. Nevertheless, these data though represent the best available HRQoL estimates for this modelled population, and no alternative utility sources were used.

## Conclusion

Surgical treatment of metastases with an ICER of 19,455€/QALY is cost-effective and below for example the NICE cost-effectiveness threshold of 24,000€–35,000€ per QALY, even in the era of the recent developments in mCRC. Metastasectomy and/or LAT should be considered whenever possible. The findings of this study can help inform healthcare decision-making concerning resource allocation and allow the cost-effectiveness of cancer therapies to be compared with other competing needs.

## Supplementary Material



## Data Availability

The model is available upon reasonable request to the corresponding author. The data collected for this study can be made available to others in a de-identified form after all primary and secondary endpoints have been published, in the presence of a data transfer agreement, and if the purpose of use complies with Finnish legislation. Requests for data sharing can be made to the corresponding author, including a proposal that must be approved by the steering committee.
