# Supplementary material for: Cost-effectiveness analysis of operative versus non-operative management of colorectal cancer metastases in the Finnish RAXO Study
Source: Acta Oncol. 2026 Feb 3;65:45005. doi: 10.2340/1651-226X.2026.45005 (PMC12881890; doi:10.2340/1651-226X.2026.45005)
Supplement: Supplementary file 1 [file AO-65-45005-s1.pdf]

Supplementary material has been published as submitted. It has not been copyedited, or typeset by Acta Oncologica

Cost-effectiveness analysis of operative versus non-operative management of colorectal cancer metastases in the Finnish RAXO Study

Joel Kontiainen[a,b], Kaisa Lehtomäki[a,b], Timo Muhonen[c], Jarmo Hahl[d], Iiro Toppila[d], Tuija Poussa[e], Emerik Osterlund[c,g], Eetu Heervä[f], Hanna Stedt[h], Raija Kallio[i], Päivi Halonen[c], Arno Nordin[j], Aki Uutela[j], Tapio Salminen[a,b], Sonja Aho[a,b], Maarit Bärlund[a,b], Annika Ålgars[f], Raija Ristamäki[f], Annamarja Lamminmäki[h], Bengt Glimelius[g], Helena Isoniemi[j*], Pia Osterlund[a,b,c,k*]

[a] Department of Oncology, TAYS Cancer Centre, Tampere University Hospital, Wellbeing Services County of Pirkanmaa, Tampere, Finland
[b] Faculty of Medicine and Health Technology, Tampere University, Tampere, Finland
[c] Department of Oncology, Helsinki University Hospital and University of Helsinki, Helsinki, Finland
[d] Medaffcon Ltd, Espoo, Finland
[e] StatConsulting, Nokia, Finland
[f] Department of Oncology, Turku University Hospital and University of Turku, Turku, Finland
[g] Department of Immunology, Genetics and Pathology, Uppsala University, Uppsala, Sweden
[h] Department of Oncology, Kuopio University Hospital and University of Eastern Finland, Kuopio, Finland
[i] Department of Oncology, Oulu University Hospital and University of Oulu, Oulu, Finland
[j] Transplantation and Liver Surgery Unit, Abdominal Centre, Helsinki University Hospital and University of Helsinki, Helsinki, Finland
[k] Department of Oncology and Pathology, Karolinska Institutet and Karolinska University Hospital, Stockholm, Sweden

Corresponding author
Pia Osterlund, M.D., PhD, prof.
Tampere University Hospital
Department of Oncology
P.O. Box 2000
30521 Tampere, Finland
Tel. +358 40 564 5653
E-mail: pia.osterlund@helsinki.fi

Supplementary Materials

Supplementary Figure 1: Study design *page 3*
Supplementary Figure 2: Model structure *page 4*
Supplementary Figure 3: The selection of the extrapolation model *page 5*
Supplementary Figure 3: Propensity score distributions by treatment group, scenario 1 *page 7*
Supplementary Figure 4: Propensity score distributions by treatment group, scenario 2 *page 8*
Supplementary Table 1: Transition probabilities *page 9*
Supplementary Table 2: Quality of life *page 11*
Supplementary Table 3: Healthcare costs *page 12*
Supplementary Table 4: Propensity score matched patient cohorts *page 13*
Supplementary Table 5: CHEERS checklist *page 14*
Supplementary Table 6: Cumulative healthcare costs and quality adjusted life-years *page 15*
Supplementary Table 7: Monte Carlo simulation summary *page 16*

References *page 17*

Supplementary Figure 1. RAXO study design

The prospective RAXO study aimed to maximize resectability of metastases. The technical resectability of liver, lung, and other metastatic sites was assessed at a specialised multidisciplinary team (MDT) meeting at Helsinki University Hospital. The patient's background and second opinion information were communicated via the raxo.fi system, developed solely for this purpose. The protocol and criteria for technical resectability, radicality of resection and outcomes, are presented in detail in the main publications from Osterlund et al Lancet Regional Health Europe 2021,[1] Isoniemi et al, Brit J Surg 2021,[2] Uutela A et al Brit J Surg 2022,[3] and Brit J Cancer 2022.[4]

Patient consent was obtained by the surgeon or oncologist at the time of metastasectomy or initiation of first systemic therapy, whether with adjuvant, neoadjuvant, conversion, or disease control indication for metastatic or locally advanced inoperable colorectal cancer. At seven large hospitals, the inclusion rate was 56% of eligible patients, and the inclusion rate at 14 smaller hospitals was 31%. According to protocol, representativity of the 1086 included patients was evaluated in comparison to data covering all 7070 mCRC patients in Finland during the same period, 2012–2018. In these comparisons, these 1086 patients were found to truly represent treatable patients in Finland according to data presented in Heervä et al ESMO GI 2023[5] and according to unpublished data (Heervä personal communication, 12th August 2025). Metastasectomy/ablation rates were higher in the prospective RAXO study than in the population-based material (37% vs 30%), but outcomes after metastasectomy/ablation or palliative systemic anti-cancer therapy (SACT) were similar. Proportions for impaired outcome features and fitness for aggressive systemic therapy were also in line (Heervä et al, personal communication 12th August 2025).

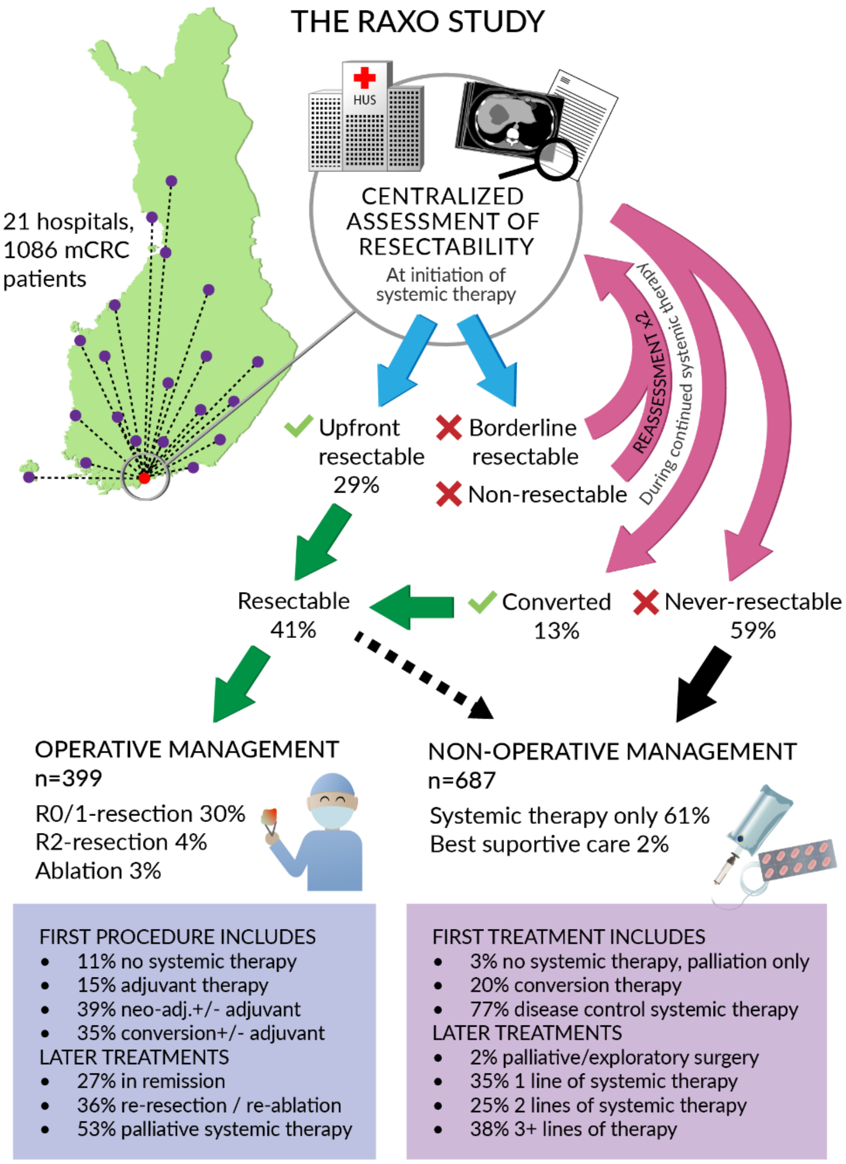

Of the 310 upfront resectable patients (representing 29% of all included patients) only 209, i.e., two-thirds, had been noted as upfront resectable even though the majority had been assessed at a local MDT. Local organ-specific surgeons or MDT meeting noted ¾ or upfront resectable whereas only ¼ was noted if assessment was done by colon MDT or rectal MDT, or by oncologists alone. [1]

Supplementary Figure 2. Markov model structure. The Markov model with input parameters is shown. The curative treatment phase is shown in green, remission in blue, and the palliative SACT phase in pink. Kaplan-Meier overall survival estimates are presented in detail in the main paper and are not directly comparable as the operative management group had more favourable baseline demographics. This was controlled for with sensitivity analyses and propensity score matching.Health-related quality-of-life was around 0.90 in the curative phase and remission, slightly lower with palliative SACT, and dropped in the end-of-life phase, details of which are presented in Supplementary Table 2.

Healthcare costs divided in six distinct treatment phases are shown and details presented in Supplementary Table 3.

Outcome measures in the model include lifetime costs, quality-adjusted life-time, and incremental cost-effectiveness ratio. BSC, best-supportive care; LAT, local ablative therapy

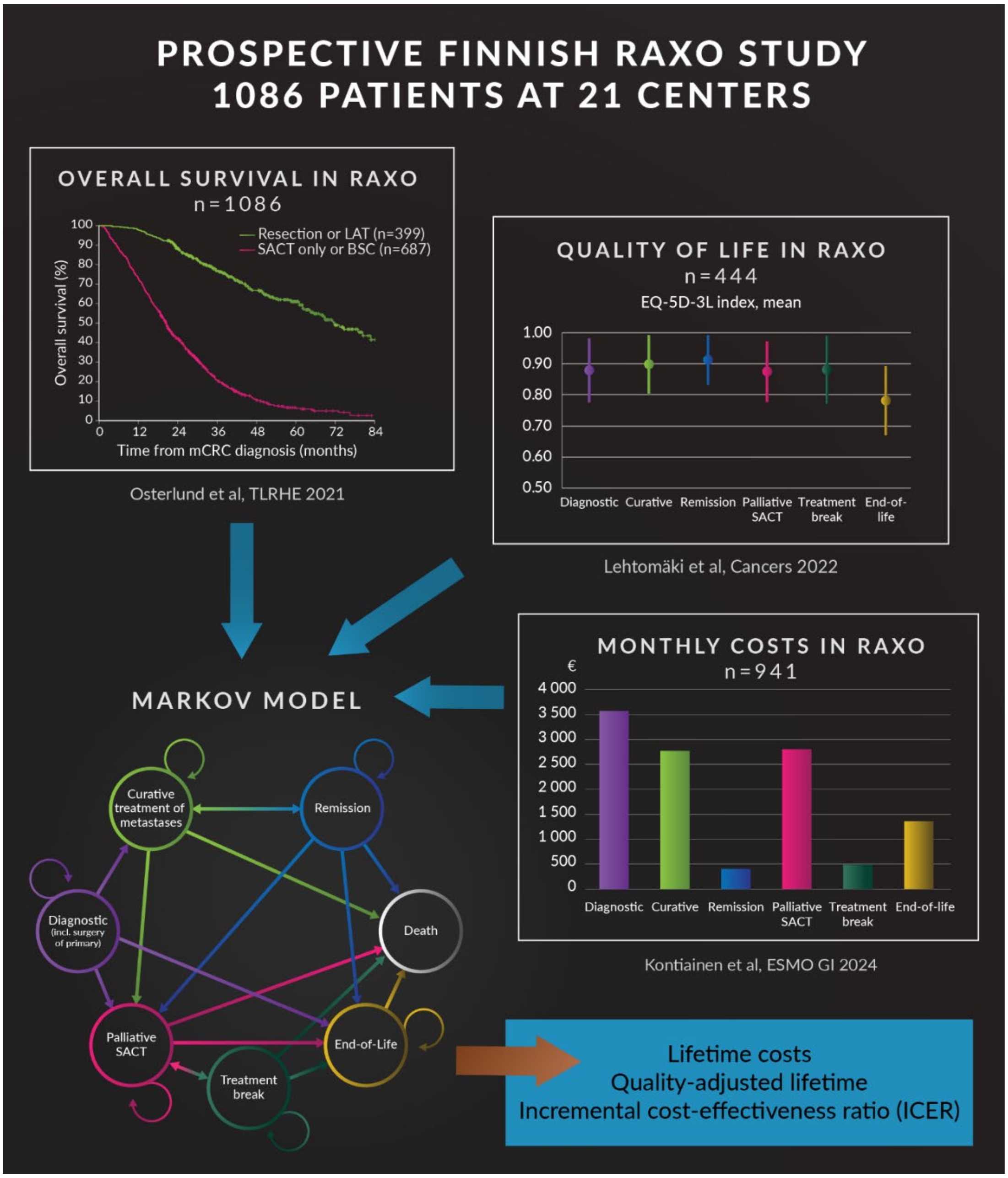

Supplementary Figure 3: Selection of the extrapolation model

In the Markov model, an exponential parametric model was used to extrapolate transition probabilities beyond the observed follow-up. Alternative parametric models were explored to assess the appropriateness of the constant hazard assumption, and the results are shown in Supplementary Figures 3A (operative treatment) and 3B (non-operative treatment). In tests based on the Akaike Information Criterion and the Bayesian Information Criterion, models allowing for non-constant hazards demonstrated superior statistical fit compared with the exponential model, indicating time-varying mortality risk. This is clinically reasonable given the elevated early risk associated with recurrence and subsequent stabilisation among long-term survivors. For example, a relapse after a curative metastasectomy mostly happens within two years after the treatment, and constant rates may underestimate long-term survival in patients in the remission health state. On the contrary, the model does not consider ageing, which increases morbidity over time.

Nevertheless, the exponential model was retained in the base-case analysis as a pragmatic and parsimonious approximation. Owing to the long follow-up of the cohort (median 94 months in the operative group and 90 months in the non-operative group), which was near-complete in the non-operative group, the contribution of extrapolation beyond the observed data was limited, and key findings were driven predominantly by empirically observed survival rather than modelled tail behaviour. As shown in Figure 1, the modelled overall survival curve with the chosen extrapolation method closely follows the estimated overall survival curve in both patient cohorts. Sensitivity analyses using different scenarios affecting survival during later health states, such as increased morbidity in the remission health state, did not markedly affect the ICER, supporting the robustness of the conclusions to the choice of parametric form. The limitations of the constant transition probabilities need to be acknowledged but are unlikely to substantially affect the interpretation of the present findings.

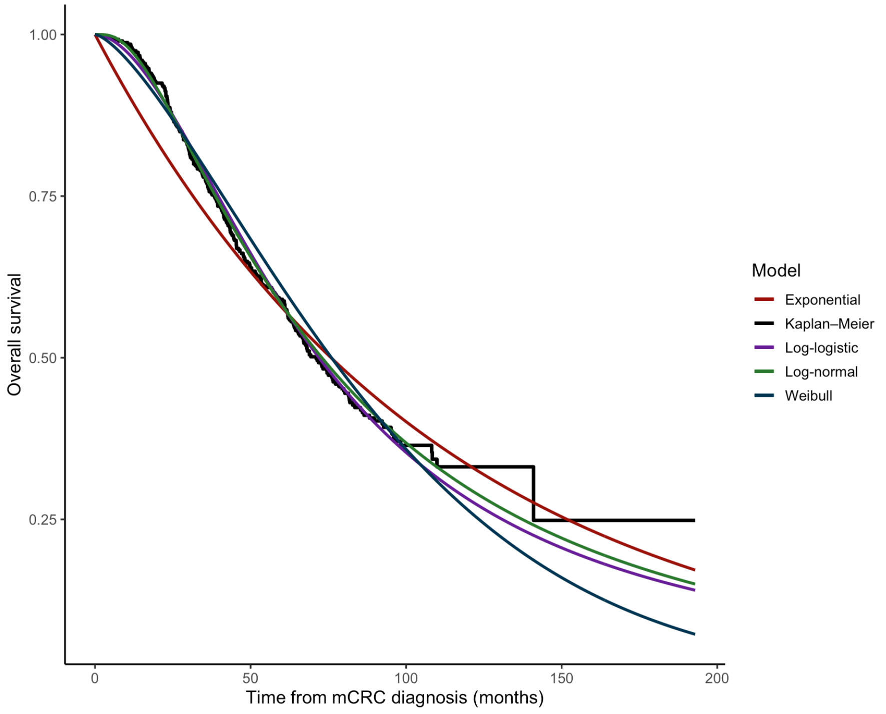

Model	AIC	BIC
Exponential	2667.6	2671.6
Weibull	2634.8	2642.8
LogNormal	2611.8	2619.7
LogLogistic	2616.2	2624.2

Supplementary Figure 3A. Comparison of extrapolation models against the overall survival Kaplan–Meier estimate in operatively treated patients

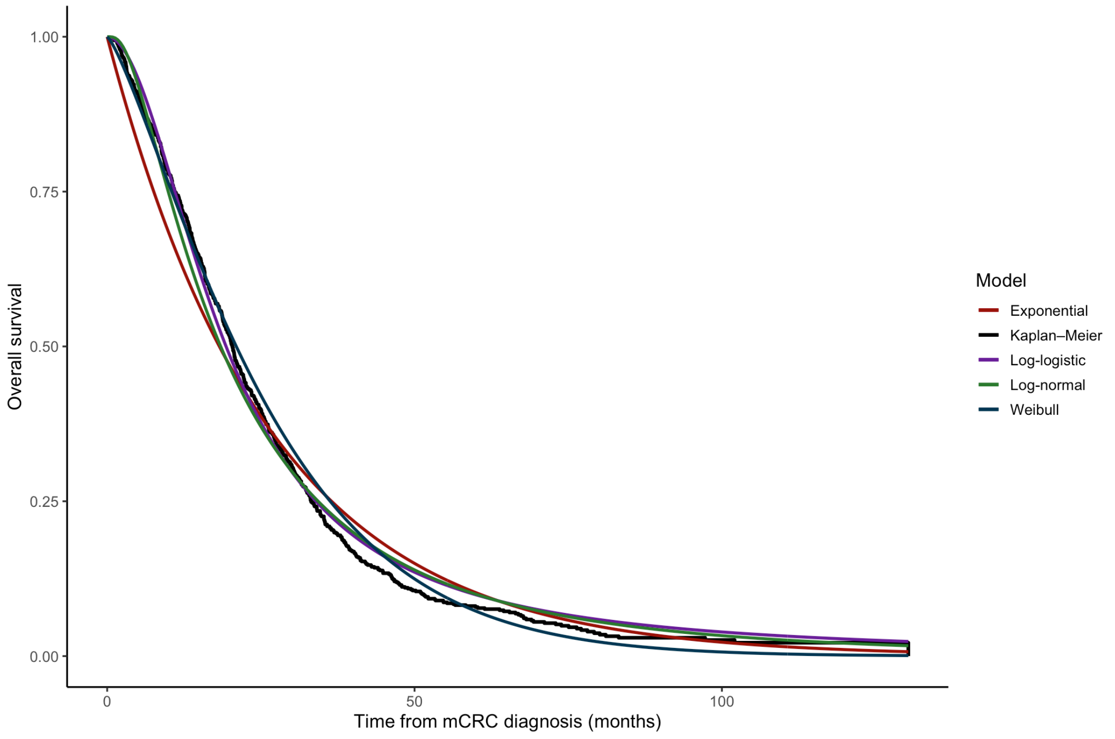

Model	AIC	BIC
Exponential	5630.6	5635.2
Weibull	5577.2	5586.3
LogNormal	5575.8	5584.9
LogLogistic	5560.8	5569.9

Supplementary Figure 3B. Comparison of extrapolation models against the overall survival Kaplan–Meier estimate in non-operatively treated patients

Supplementary Figure 4. Boxplot of the propensity scores for operative and non-operative groups, before and after propensity score matching. The propensity scores were estimated using a multivariable logistic model and the following covariates: ECOG, primary location, metastatic sites, mutational status, upfront resectability by central assessment and age. The boxplot presents the 10th percentile, the 25th percentile, the median, the 75th percentile and the 90th percentile.

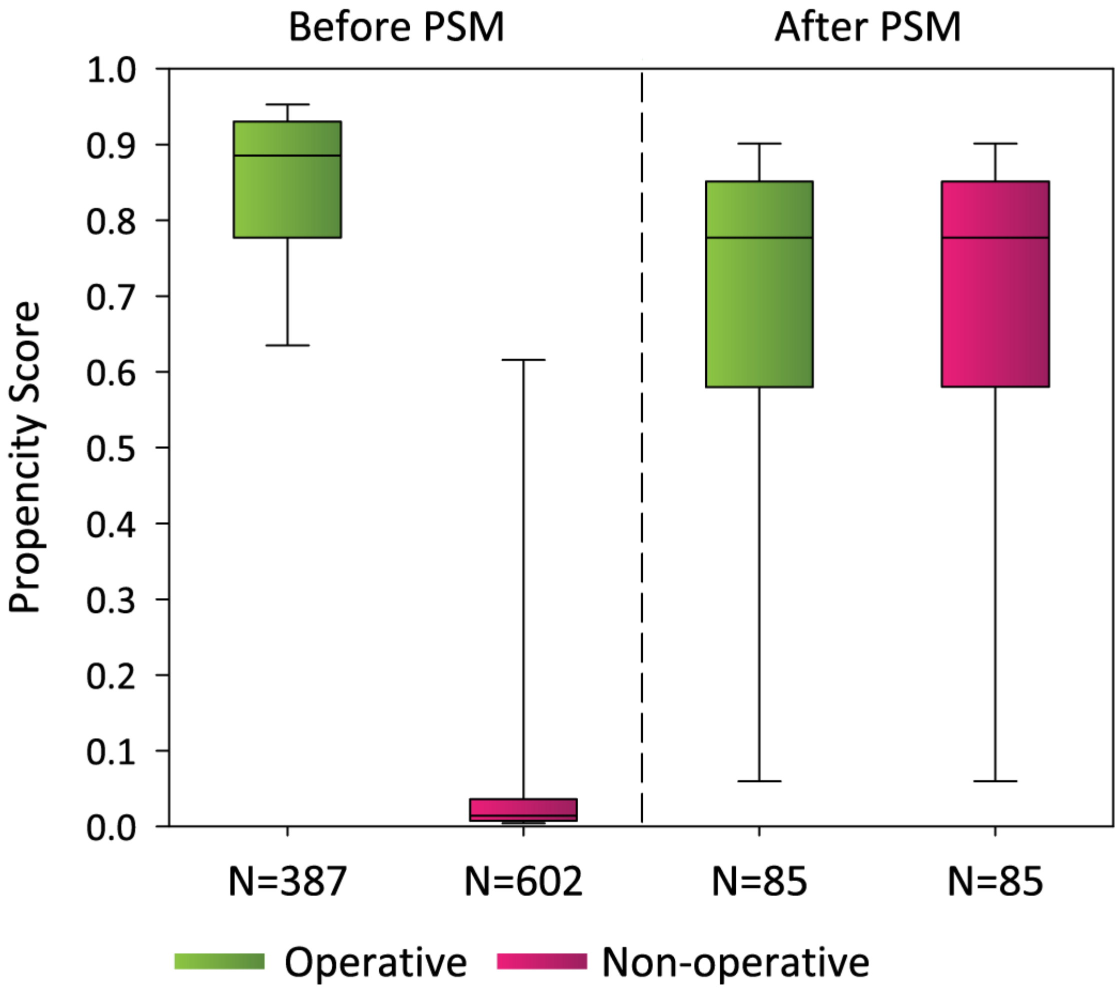

Supplementary Figure 5. Boxplot of the propensity scores for operative and non-operative groups, before and after propensity score matching. The propensity scores were estimated using a multivariable logistic model and the following covariates: ECOG, primary location, metastatic sites and mutational status. The boxplot presents the 10th percentile, the 25th percentile, the median, the 75th percentile and the 90th percentile.

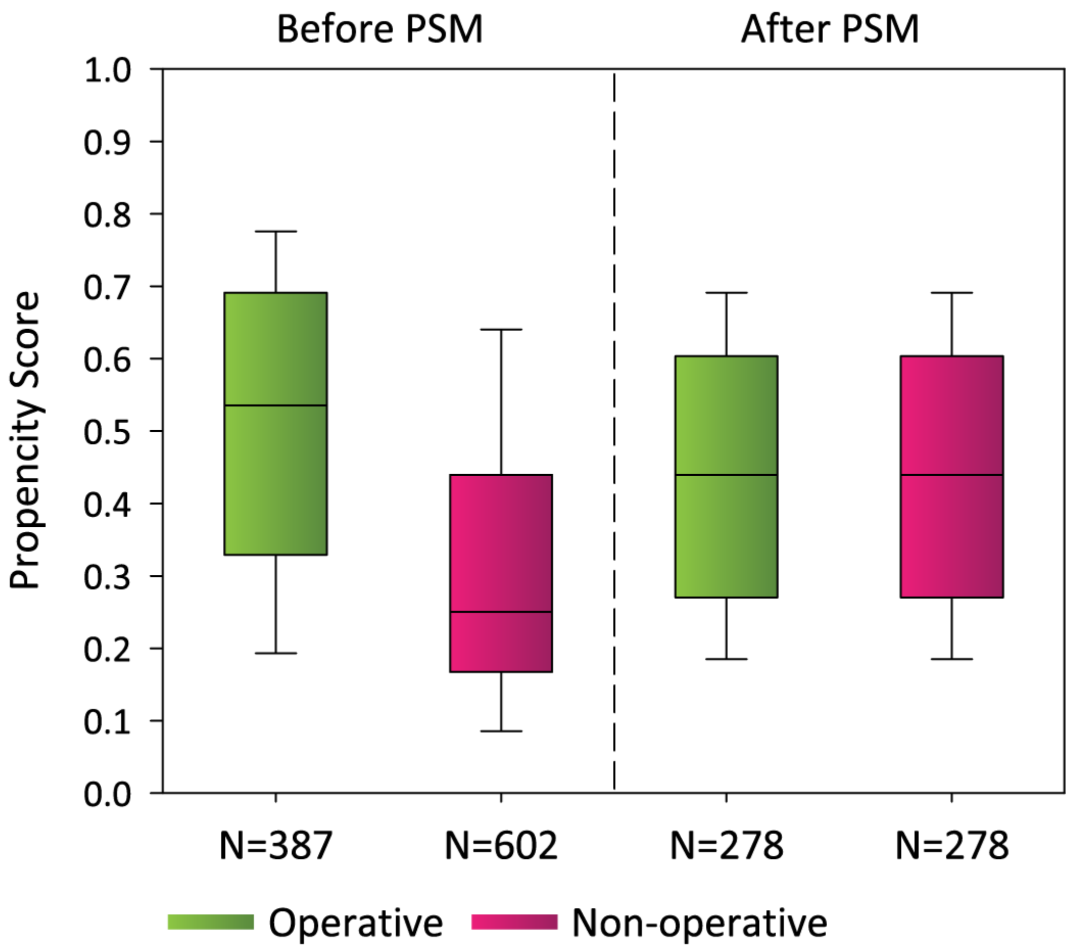

Supplementary Table 1a: Transition probabilities

Cohort	Health state A	n (patients)	n (transitions)	Health state B	Transition probability A->B	SD	Distribution
Operative	Diagnostic	374	831	Diagnostic	0.537	0.231	Beta
				Curative	0.463	0.272	
	Curative	399	8 444	Curative	0.942	0.073	Dirichlet
				Remission	0.039	0.058	
				Palliative SACT	0.015	0.038	
				Treatment break	0.001	0.008	
				End-of-life	0.000	0.003	
				Death	0.002	0.014	
	Remission	278	11 031	Curative	0.008	0.037	Dirichlet
				Remission	0.979	0.065	
				Palliative SACT	0.008	0.037	
				Treatment break	0.000	0.002	
				End-of-life	0.001	0.006	
				Death	0.004	0.033	
	Palliative SACT	199	4 236	Palliative SACT	0.962	0.062	Dirichlet
				Treatment break	0.014	0.030	
				End-of-life	0.013	0.029	
				Death	0.010	0.035	
	Treatment break	80	773	Treatment break	0.906	0.174	Dirichlet
				End-of-life	0.083	0.168	
				Death	0.011	0.062	
	End-of-life	124	298	End-of-life	0.582	0.204	Beta
				Death	0.418	0.204	
Non-operative	Diagnostic	677	1 398	Diagnostic	0.533	0.230	Dirichlet
				Palliative SACT	0.461	0.248	
				End-of-life	0.001	0.032	
				Death	0.005	0.056	
	Palliative SACT	673	13 180	Palliative SACT	0.950	0.066	Dirichlet
				Treatment break	0.012	0.030	
				End-of-life	0.017	0.043	
				Death	0.021	0.057	
	Treatment break	170	1 865	Treatment break	0.914	0.168	Dirichlet
				End-of-life	0.075	0.156	
				Death	0.011	0.072	
	End-of-life	359	776	End-of-life	0.535	0.234	Beta
				Death	0.465	0.234	

SD, standard deviation; SACT, systemic anti-cancer therapy

Supplementary Table 1b: Transition probability matrix

		Diagnostic	Curative	Remission	Palliative SACT	Treatment Break	End-of-life	Death	SUM
Operative	Diagnostic	0.537	0.463	0.000	0.000	0.000	0.000	0.000	1.000
	Curative	0.000	0.942	0.039	0.015	0.001	0.000	0.002	1.000
	Remission	0.000	0.008	0.979	0.008	0.000	0.001	0.004	1.000
	Palliative SACT	0.000	0.000	0.000	0.962	0.014	0.013	0.010	1.000
	Treatment Break	0.000	0.000	0.000	0.000	0.906	0.083	0.011	1.000
	End-of-life	0.000	0.000	0.000	0.000	0.000	0.582	0.418	1.000
Non-operative	Diagnostic	0.533	0.000	0.000	0.461	0.000	0.001	0.005	1.000
	Palliative SACT	0.000	0.000	0.000	0.950	0.012	0.017	0.021	1.000
	Treatment Break	0.000	0.000	0.000	0.000	0.914	0.075	0.011	1.000
	End-of-life	0.000	0.000	0.000	0.000	0.000	0.535	0.465	1.000

Supplementary Table 2: Health-related quality-of-life according to EQ-5D-3L in 444 RAXO patients

EQ-5D-3L, TTO	Diagnostic	Curative	Remission	Palliative SACT	Treatment break	End-of-life
Number of patients answered	21	114	145	256	37	12
Number of patients with ≥1 complete questionnaire	14	104	141	216	37	12
Questionnaires returned, count*	21	317	447	889	88	12
Questionnaires with missing values, count	7	51	18	207	6	0
Questionnaires with missing values, %	33 %	16 %	4 %	23 %	7 %	0 %
Complete questionnaires used in HRQoL estimation	14	266	429	682	82	12
EQ-5D-3L index, mean	0.88	0.90	0.91	0.88	0.88	0.78
EQ-5D-3L index, SD	0.10	0.09	0.08	0.10	0.11	0.11

*Multiple questionnaires per patient were permitted.
HRQoL estimates are based on complete EQ-5D-3L profiles.
A gamma distribution was assumed for the HRQoL parameters used as model inputs.
TTO, time trade off; SD, standard deviation; SACT, systemic anti-cancer therapy; HRQoL, health-related quality of life

Complete EQ-5D index data were available for 84% of the 1774 questionnaires, while 289 (16%) were missing due to incomplete or invalid EQ-5D profiles. The index score cannot be calculated if answers are missing. In line with the previous RAXO publication from which the QoL data were derived[6], no imputation was used for EQ-5D values.

Supplementary Table 3. Healthcare costs in six distinct treatment phases

	Diagnostic		Curative		Remission		Palliative SACT		Treatment break		End-of-life		
	Mean, €	SD, €	Mean, €	SD, €	Mean, €	SD, €	Mean, €	SD, €	Mean, €	SD, €	Mean, €	SD	Reference
Total hospital costs PPPM in 2021 price level	3 662	6 444	3 059	1 732	453	1 039	2 963	1 641	560	1 348	1 695	3 011	28
Adjustment for inflation to 2023 price level	+ 359	+ 632	+ 300	+ 170	+ 44	+ 102	+ 290	+ 161	+ 55	+ 132	+ 166	+ 295	45
Primary care costs in 2023 price level*	+ 103	-	+ 103	-	+ 95	-	+ 103	-	+ 97	-	+ 1341	-	29
Outpatient chemotherapy in 2023 price level*	-	-	+ 29	-	-	-	+ 125	-	-	-	-	-	28
Total	4 124	7 076	3 491	1 901	592	1 141	3 481	1 802	711	1 480	3 203	3 307	

*Calculated by summing the costs from the reference and adjusting them for inflation to the 2023 price level
A gamma distribution was assumed for the healthcare cost parameters used as model inputs.
PPPM, Per Patient Per Month; SD, standard deviation, SACT systemic anti-cancer therapy

Supplementary Table 4: Propensity score matched patient cohorts

			Original groups					Propensity score matched subgroup, scenario 1 (caliper radius = 0.2*sigma)					Propensity score matched subgroups, scenario 2 (caliper radius = 0.2*sigma)				
			N = 1086					N = 170					N = 556				
			Operative		Non-operative			Operative		Non-operative			Operative		Non-operative		
			n = 399	%	n = 687	%	SMD	n = 85	%	n = 85	%	SMD	n = 278	%	n = 278	%	SMD
Covariates used for propensity score matching	Age*	median (range)	65	(25.0–83.6)	67.4	(24.3–89.7)		67.5	(41.9–83.6)	68.7	(46.2–85.7)		65.5	(25.0–83.6)	66.2	(31.0–85.7)	
	Age*	≤70	290	73 %	425	62	0.23	52	61	51	60	0.02	196	71	183	66	0.10
		>70	109	27	262	38		33	39	34	40		82	29	95	34	
	ECOG	PS 0	159	40	136	20	0.45	14	16	17	20	-0.09	75	27	73	26	0.02
		PS 1	210	53	390	57	-0.08	61	72	56	66	0.13	174	63	176	63	-0.01
		PS 2-3	30	8	161	23	-0.45	10	12	12	14	-0.07	29	10	29	10	0.00
	Primary location	Right colon	86	22	224	33	-0.25	21	25	26	31	-0.13	65	23	67	24	-0.02
		Left colon	176	44	220	32	0.25	28	33	38	45	-0.24	116	42	103	37	0.10
		Rectum	137	34	237	34	0.00	36	42	21	25	0.38	97	35	108	39	-0.08
		Multiple	0	0.00	6	0.90	-0.13	0	0	0	0	0.00	0	0	0	0	0.00
	Metastatic sites	1 site	309	77	277	40	0.81	55	65	55	65	0.00	190	68	190	68	0.00
		2 sites	62	16	257	37	-0.51	21	25	24	28	-0.08	60	22	60	22	0.00
		3 to 6 sites	28	7	153	22	-0.44	9	11	6	7	0.12	28	10	28	10	0.00
	Mutational status	RAS +/- BRAF wt	175	44	254	37	0.14	29	34	30	35	-0.02	77	28	85	31	-0.06
		RAS mt	200	50	343	50	0.00	52	61	51	60	0.02	155	56	156	56	-0.01
		BRAF mt	16	4	78	11	-0.28	4	5	4	5	0.00	20	7	19	7	0.01
		Not tested	8	2	12	2	0.02	0	0	0	0	0.00	26	9	18	6	0.11
	Upfront resectability by central assessment*	Upfront resectable/neoadjuvant	265	66	45	7	1.58	34	40	39	46	-0.12	179	64	26	9	1.39
		Borderline/conversion	125	31	54	8	0.62	42	49	37	44	0.12	92	33	28	10	0.58
		Non-resectable	9	2	588	86	-3.09	9	11	9	11	0.00	7	3	224	81	-2.59
Other baseline characteristics	Sex	Male	242	61	414	60	0.01	46	54	57	67	-0.27	160	58	179	64	-0.14
		Female	157	39	273	40		39	46	28	33		118	42	99	36	
	Charlson comorbidity index	No	320	80	514	75	0.13	71	84	63	24	1.51	223	80	203	73	0.17
		1 to 2	77	19	167	24	-0.12	13	15	20	2	0.47	54	19	71	26	-0.15
		3 to 5	2	0.50	6	0.90	-0.05	1	1	2	2	-0.09	1	0	4	1	-0.11
	Smoking status	Never smoker	160	57	244	50	0.14	31	62	29	45	0.34	114	58	102	50	0.18
		Ex-smoker	83	30	178	36	-0.15	11	22	23	36	-0.31	54	28	69	34	-0.13
		Smoker	38	14	68	14	-0.01	8	16	12	19	-0.07	27	14	34	17	-0.08
	BMI	< 20	31	8	53	8	0.00	3	4	6	7	-0.16	24	9	15	5	0.13
		20-30	278	70	524	76	-0.15	62	73	68	80	-0.17	188	68	214	77	-0.21
		≥ 30	90	23	110	16	0.17	20	24	11	13	0.28	66	24	49	18	0.15
	Surgery of primary ever	Upfront	324	81	401	58	0.51	62	73	69	81	-0.20	220	79	175	63	0.36
		During	69	17	38	6	0.38	21	25	5	6	0.54	53	19	23	8	0.32
		No	6	2	248	36	-0.99	2	2	11	13	-0.41	5	2	80	29	-0.81
	Presentation of metastases	Synchronous	231	58	505	74	-0.33	57	67	53	62	0.10	165	59	195	70	-0.23
		Metachronous	168	42	182	26		28	33	32	38	-0.10	113	41	83	30	0.23
	Mismatch repair status	Proficient (MSS)	229	57	253	37	0.42	51	60	38	45	0.31	143	51	128	46	0.11
		Deficient (MSI-H)	8	2	10	1.50	0.04	1	1	1	1	0.00	5	2	4	1	0.03
		Not tested	162	41	424	62	-0.43	33	39	46	54	-0.31	130	47	146	53	-0.12

*Propensity score matching was in scenario 1 performed based on ECOG, number of metastatic sites, mutational status, primary tumour location, resectability (upfront, borderline, or nonresectable), and age. In scenario 2, matching was done with same parameters excluding resectability and age.

Supplementary Table 5: CHEERS check

List Item		Guidance for Reporting	Reported in section
**TITLE**			
Title	1	Identify the study as an economic evaluation and specify the interventions being compared.	Title
**ABSTRACT**			
Abstract	2	Provide a structured summary that highlights context, key methods, results and alternative analyses.	Abstract
**INTRODUCTION**			
Background and objectives	3	Give the context for the study, the study question and its practical relevance for decision making in policy or practice.	Introduction
**METHODS**			
Health economic analysis plan	4	Indicate whether a health economic analysis plan was developed and where available.	Methods, section 1
Study population	5	Describe characteristics of the study population (such as age range, demographics, socioeconomic, or clinical characteristics).	Results, section 1
Setting and location	6	Provide relevant contextual information that may influence findings.	Introduction, discussion section 4–5
Comparators	7	Describe the interventions or strategies being compared and why chosen.	Introduction section 2, Methods section 3
Perspective	8	State the perspective(s) adopted by the study and why chosen.	Introduction section 4, Methods section 1 and 7
Time horizon	9	State the time horizon for the study and why appropriate.	Introduction section 4, Methods section 1
Discount rate	10	Report the discount rate(s) and reason chosen.	Methods section 8
Selection of outcomes	11	Describe what outcomes were used as the measure(s) of benefit(s) and harm(s).	Methods section 1
Measurement of outcomes	12	Describe how outcomes used to capture benefit(s) and harm(s) were measured.	Methods section 6–7
Valuation of outcomes	13	Describe the population and methods used to measure and value outcomes.	Methods section 6–7
Measurement and valuation of resources and costs	14	Describe how costs were valued.	Methods section 7
Currency, price date, and conversion	15	Report the dates of the estimated resource quantities and unit costs, plus the currency and year of conversion.	Methods section 7
Rationale and description of model	16	If modelling is used, describe in detail and why used. Report if the model is publicly available and where it can be accessed.	Methods section 1
Analytics and assumptions	17	Describe any methods for analysing or statistically transforming data, any extrapolation methods, and approaches for validating any model used.	Methods section 9–12
Characterizing heterogeneity	18	Describe any methods used for estimating how the results of the study vary for sub-groups.	Methods section 10
Characterizing distributional effects	19	Describe how impacts are distributed across different individuals or adjustments made to reflect priority populations.	Not reported
Characterizing uncertainty	20	Describe methods to characterize any sources of uncertainty in the analysis.	Methods section 10–11
Approach to engagement with patients and others affected by the study	21	Describe any approaches to engage patients or service recipients, the general public, communities, or stakeholders in the design of the study.	Not reported
**RESULTS**			
Study parameters	22	Report all analytic inputs (e.g., values, ranges, references) including uncertainty or distributional assumptions.	Tables S1–S3
Summary of main results	23	Report the mean values for the main categories of costs and outcomes of interest and summarise them in the most appropriate overall measure.	Table 2
Effect of uncertainty	24	Describe how uncertainty about analytic judgments, inputs, or projections affect findings. Report the effect of choice of discount rate and time horizon, if applicable.	Table 3, Results section 6–9, Discussion section 5–6
Effect of engagement with patients and others affected by the study	25	Report on any difference patient/service recipient, general public, community, or stakeholder involvement made to the approach or findings of the study	Not reported
**DISCUSSION**			
Study findings, limitations, generalizability, and current knowledge	26	Report key findings, limitations, ethical or equity considerations not captured, and how these could impact patients, policy, or practice.	Discussion section 5–6
**OTHER RELEVANT INFORMATION**			
Source of funding	27	Describe how the study was funded and any role of the funder in the identification, design, conduct, and reporting of the analysis	Funding and conflict of interests statements
Conflicts of interest	28	Report authors conflicts of interest according to journal or International Committee of Medical Journal Editors requirements.	Funding and conflict of interests statements

Supplementary Table 6. Cumulative healthcare costs and quality adjusted life-years

	Costs						QALY			
	Operative			Non-operative			Operative		Non-operative	
Years from diagnosis	Cost / year	Cumulative cost	cumulative %	Cost / year	Cumulative cost	cumulative %	QALY/ year	Cumulative QALY	QALY / year	Cumulative QALY
0	- €	- €	0	- €	- €	0	0.00	0.00	0.00	0.00
1	38 370 €	38 370 €	24	36 738 €	36 738 €	48	0.89	0.89	0.76	0.76
2	22 673 €	62 784 €	40	18 899 €	55 636 €	72	0.72	1.67	0.44	1.20
3	17 940 €	80 724 €	51	10 151 €	65 787 €	85	0.68	2.35	0.25	1.45
4	13 881 €	94 605 €	60	5 400 €	71 187 €	92	0.58	2.93	0.14	1.58
5	11 083 €	105 688 €	67	2 855 €	74 042 €	96	0.49	3.42	0.07	1.65
6	9 013 €	113 321 €	72	1 673 €	75 357 €	98	0.41	3.77	0.04	1.69
7	7 408 €	122 109 €	77	789 €	76 335 €	99	0.34	4.18	0.02	1.71
8	6 126 €	128 235 €	81	414 €	76 749 €	99	0.29	4.46	0.01	1.72
9	5 084 €	133 319 €	84	217 €	76 965 €	100	0.24	4.70	0.01	1.73
10	4 228 €	137 548 €	87	113 €	77 079 €	100	0.20	4.90	0.00	1.73
11	3 521 €	141 069 €	89	59 €	77 138 €	100	0.17	2.14	0.00	1.38
12	2 935 €	144 003 €	91	31 €	77 169 €	100	0.14	5.21	0.00	1.74
13	2 447 €	146 450 €	93	16 €	77 185 €	100	0.12	5.33	0.00	1.74
14	2 041 €	148 491 €	94	8 €	77 193 €	100	0.10	5.42	0.00	1.74
15	1 703 €	150 194 €	95	4 €	77 198 €	100	0.08	5.50	0.00	1.74
16	1 421 €	151 616 €	96	2 €	77 200 €	100	0.07	5.57	0.00	1.74
17	1 186 €	152 802 €	97	1 €	77 201 €	100	0.06	5.63	0.00	1.74
18	990 €	153 792 €	97	1 €	77 202 €	100	0.05	5.67	0.00	1.74
19	826 €	154 618 €	98	0 €	77 202 €	100	0.04	5.71	0.00	1.74
20	690 €	155 307 €	98	0 €	77 202 €	100	0.03	5.75	0.00	1.74

QALY, quality-adjusted life-year; SD, standard deviation

Supplementary Table 7: Monte Carlo simulation summary

	Healthcare costs, €		QALY		Life years	
Monte Carlo simulation	Operative	Non-operative	Operative	Non-operative	Operative	Non-operative
Sample size (n)	10 000	10 000	10 000	10 000	10 000	10 000
Mean	158 964	77 228	5.92	1.74	6.60	1.99
SD of the mean	67 014	35 736	0.42	0.16	0.28	0.06
Minimum	24 736	6 199	3.93	0.94	5.62	1.76
Median	147 048	71 789	5.95	1.77	6.59	1.99
Maximum	757 328	328 463	7.41	2.15	7.81	2.22
95% CI lower bound of the mean	157 651	76 528	5.91	1.74	6.59	1.99
95% CI upper bound of the mean	160 278	77 929	5.93	1.74	6.60	2.00

QALY, quality-adjusted life-year; SD, standard deviation; CI, confidence interval

References

1. Osterlund P, Salminen T, Soveri LM, Kallio R, Kellokumpu I, Lamminmäki A, *et al.* Repeated centralized multidisciplinary team assessment of resectability, clinical behavior, and outcomes in 1086 Finnish metastatic colorectal cancer patients (RAXO): A nationwide prospective intervention study. *Lancet Reg Health Eur* 2021;**3**:100049.
2. Isoniemi H, Uutela A, Nordin A, et al. Centralized repeated resectability assessment of patients with colorectal liver metastases during first-line treatment: prospective study. *Br J Surg* 2021; **108**(7):817-25.
3. Uutela A, Osterlund E, Halonen P, et al. Resectability, conversion, metastasectomy, and outcome accroding to RAS and BRAF status for metastatic colorectal cancer in the prospective RAXO study. *Brit J Cancer* 2022; **127**(4): 686-94.and Brit J Cancer 2022.[4]
4. Uutela A, Nordin A, Osterlund E, et al. Resectability and resection rates of colorectal liver metastases according to RAS and BRAF mutational status: prospective study. *Brit J Surg* 2023; **110**(8): 931-5
5. Heervä E, Ristimäki A, Kytölä S, Halonen P, Soveri L, Salminen T, et al. PD-19 Finnish population-based metastatic colorectal cancer data collection study – comparison with the prospective RAXO study. Ann Oncol 2023;**34**:S8–S9.
6. Lehtomäki K, Stedt HP, Osterlund E, et al. Health-Related Quality of Life in Metastatic Colorectal Cancer Patients Treated with Curative Resection and/or Local Ablative Therapy or Systemic Therapy in the Finnish RAXO-Study. Cancers. 2022;14(7).
